# HPV self-sampling as an additional option in cervical cancer screening: a pilot study in Estonia

**DOI:** 10.1093/eurpub/ckac130.004

**Published:** 2022-10-25

**Authors:** R Hallik, K Innos, J Jänes, P Veerus

**Affiliations:** Epidemiology and Biostatistics, The National Institute for Health Development, Tallinn, Estonia

## Abstract

**Background:**

Cervical cancer incidence and mortality rates remain high in Estonia and participation in organized cervical cancer screening program is low. The aim of this pilot study was to estimate the impact of offering an HPV self-sampling option on screening uptake.

**Methods:**

A randomized intervention study was conducted within Estonian organized cervical cancer screening program in 2021. Among target group women who had not participated in screening by August 2021, 26,000 women were randomly selected and allocated to two equally sized intervention arms offering a choice between attending a clinic or taking a self-sample. The opt-out group received a Qvintip sampler by regular mail to home address, the opt-in group received by e-mail a link to order the sampler from a web-site. A control group of 32,000 women received the usual reminder to attend screening at a clinic. Participation rates were calculated and data on user experience were collected with a questionnaire.

**Results:**

Significant difference in participation rates was observed between opt-out (41%) (among them 20% chose self-sampling, 21% chose clinic attendance), opt-in (34%) (8% self-sampling, 26% clinic) and control group (28%). Intervention arms showed higher screening uptake in all age-groups and regions, but the largest effect was seen at ages 60 and 65 years and in regions showing the lowest screening participation rates. Among self-sampling users, 99% agreed that self-sampling was easy and only 3% prefer testing at a clinic.

**Conclusions:**

Offering women a choice between HPV self-sampling or attending a clinic significantly increased cervical cancer screening uptake. Sending an HPV self-sampling kit to home address was the most effective approach. Majority of women who chose HPV self-sampling want to use this option in the future. HPV self-sampling should be integrated in the cervical cancer screening program in Estonia.

**Key messages:**

- HPV self-sampling increased cervical cancer screening participation in Estonia and was highly accepted.
- It is important to make HPV self-sampling available in cervical cancer screening program.

